# Carbon dioxide: the cause of devastating stroke without hemodynamic compromise during laparoscopic nephrectomy with injury of the inferior vena cava: A case report

**DOI:** 10.1097/MD.0000000000024892

**Published:** 2021-02-26

**Authors:** Youxiu Yao, Mao Xu

**Affiliations:** Department of Anesthesiology, Peking University Third Hospital, Beijing, P.R. China.

**Keywords:** carbon dioxide, cerebral infarction, delayed recovery, paradoxical embolism, patent foramen ovale

## Abstract

**Rationale:**

Carbon dioxide pneumoperitoneum in laparoscopic surgery can bring about occult perioperative cerebral infarction, advancing our understanding of the causes of severe postoperative delayed recovery.

**Patient concerns:**

Here, we report the case of a 35-year-old woman who underwent a right renal tumor resection in our institution, during which a raised pneumoperitoneum pressure (from 15 to 20 mm Hg) was adopted by the surgeon to prevent errhysis and to help stop the bleeding. Despite an accidental minor tearing of the inferior vena cava, vital signs remained stable throughout the procedure, and no obvious abnormality was observed in either end tidal carbon dioxide values or blood gas analysis. However, the patient unexpectedly suffered delayed recovery after the operation, presenting incomplete left hemiplegia and a positive Babinski sign.

**Diagnoses:**

Perioperative stroke was diagnosed by anesthesiologists, after excluding the effects of anesthesia. Cerebral hemorrhage was excluded, as no obvious abnormality was found in the density of brain parenchyma in the emergency computed tomography examination, and a digital subtraction angiography showed no abnormal thrombosis. Further magnetic resonance diagnosis led us to consider diffuse gas embolisms to be the cause of this acute stroke; a right echocardiography revealed that a patent foramen ovale (PFO) may account for the global cerebral gas embolisms.

**Interventions:**

The patient received neuroprotective drugs (Vinpocetine, Edaravone, and Xingnaojing, which are commonly used as a standard of care in China), antiplatelets and other symptomatic treatments, plus dexamethasone to relieve edema. A contrast-enhanced echocardiography of the right heart was performed, the results of which were consistent with the sonography of a PFO.

**Outcomes:**

The patient was hospitalized for 14 days and eventually discharged after recovery. At the latest follow-up in August 2019, the patient recovered without residual neurological sequelae.

**Lessons:**

Our results emphasize the need for vigilance regarding adverse cardiovascular and neurological events caused by carbon dioxide gas embolisms when encountering the inadvertent situation of vessels rupturing. Timely monitoring of paradoxical gas embolism by transoesophageal echocardiography is necessary and can avert the risk of severe complications. Urgent consideration should be given to stopping pneumoperitoneum and switching to laparotomy for hemostasis so that the patient can obtain the best benefit–risk ratio.

## 1 Introduction

Laparoscopic tumor resection is becoming increasingly popular among patients and surgeons due to its minimal invasiveness, clear vision, and fast postoperative recovery. Carbon dioxide is generally used for pneumoperitoneum, which is safe because of its high solubility in the blood, and its tendency not to produce gas embolism. Only when a large amount of carbon dioxide enters the circulatory system will embolism occur, with a very low incidence of about 0.15%.^[1]^ In the process of tumor separation, occasional small lacerations of blood vessels are inevitable, resulting in a blurred visual field due to oozing blood. At such moments, is it appropriate to use pneumoperitoneum pressure to stop the bleeding and to continue the operation via laparoscopy? According to research reports, the first response of surgeons is that a certain degree of pneumoperitoneum pressure can help stop the bleeding,^[2–4]^ but this will in turn increase the incidence of gas embolism^[5]^ and also increase the complexity of perioperative management. Under these circumstances, carbon dioxide will be absorbed into the blood at pneumoperitoneum pressure, which will not only cause subcutaneous emphysema and delayed recovery (carbon dioxide anesthesia), but also damage to important organs, for example, heart, lungs, and brain, and even occasionally pulmonary embolism or paradoxical gas embolism.^[6]^ This kind of gas embolism complication is rare but usually fatal, with a high probability of cardiac arrest,^[7]^ which is usually accompanied by hemodynamic instability, a sharp drop in blood pressure, and a decrease^[8]^ in end tidal carbon dioxide (ETCO_2_) values. However, paradoxical gas embolism causing cerebral infarction in laparoscopic surgery may still occur without observable abnormalities in blood pressure, CO_2_, and blood gas analysis, which is even rarer; to date no relevant report exists of such a case. Here, we report a case of occult cerebral infarction during retroperitoneal laparoscopic renal tumor resection with a minor tearing of the inferior vena cava, which was only identified due to severe delayed recovery.

## 2 Case presentation

The report was approved by the ethics review board of Peking University Third Hospital and was CARE compliant. Informed consent for publication was obtained from the patient.

The patient, a 35-year-old female (height 165 cm, weight 64 kg), was admitted to hospital due to flank pain persisting for more than 1 month, and was diagnosed with right renal carcinoma. It was therefore proposed to perform a laparoscopic right renal tumor resection under general anesthesia. The patient's previous medical history includes appendectomy and ectopic pregnancy surgery, and penicillin allergy. She had no communicable diseases, for example, hepatitis, tuberculosis, malaria; no history of hypertension, diabetes, and cardiac or cerebrovascular diseases; and no episodes of mental illness, trauma, blood transfusions, or food allergies. All preoperative evaluation tests, including routine blood, routine coagulation function, liver and kidney function, Electrocardiogram (ECG) and chest radiograph, were unremarkable. General anesthesia was induced using sufentanil 30 μg, etomidate 12 mg, propofol 80 mg, and rocuronium 50 mg, monitoring pulse oxygen saturation, capnography, ECG and invasive arterial blood pressure, and was maintained using a mixture of O_2_ 1 L/min, air 1 L/min, sevoflurane 1.5 to 2.0 vol %, and remifentanil 0.05 to 0.2 μg/kg/min. The left lateral decubitus position is employed to facilitate the surgery.

Thirty minutes after the beginning of the surgery, the pneumoperitoneum pressure was increased (15–20 mm Hg) to help stop bleeding. At this point, ETCO_2_ was 41 mm Hg. During the procedure, an accidental minor tearing of the inferior vena cava occurred. A gauze compression was used to stop the bleeding, and the rupture was sutured as quickly as possible. ETCO_2_ (41–47 mm Hg) and invasive blood pressure were stable (118–145/65–79 mm Hg) during the process, and there were no obvious abnormal observations, except for the heart rate, which had a period of fluctuation (range 45–120 times/min); but then returned to normal with no specific treatment. Arterial blood gas analysis: pH = 7.30, PaCO_2_ = 48 mm Hg, PaO_2_ = 455 mm Hg, K^+^ = 3.2 mmol/L, Ca^2+^ = 1.13 mmol/L, HCO_3_^-^ = 21.1 mmol/L, Glucose = 4.2 mmol/L, Base Excess = −3.8 mmol/L, Hb = 125 g/L, SaO_2_ = 100%. Anesthesiologists provided potassium supplement, increased the respiration rate of mechanical ventilation, and continued to observe without any further treatment. Subsequently, all indicators were stable and the operation was successfully completed. The bleeding volume was 50 mL, the urine output and the total input volumes were 100 and 2600 mL, respectively.

**Figure 1:**
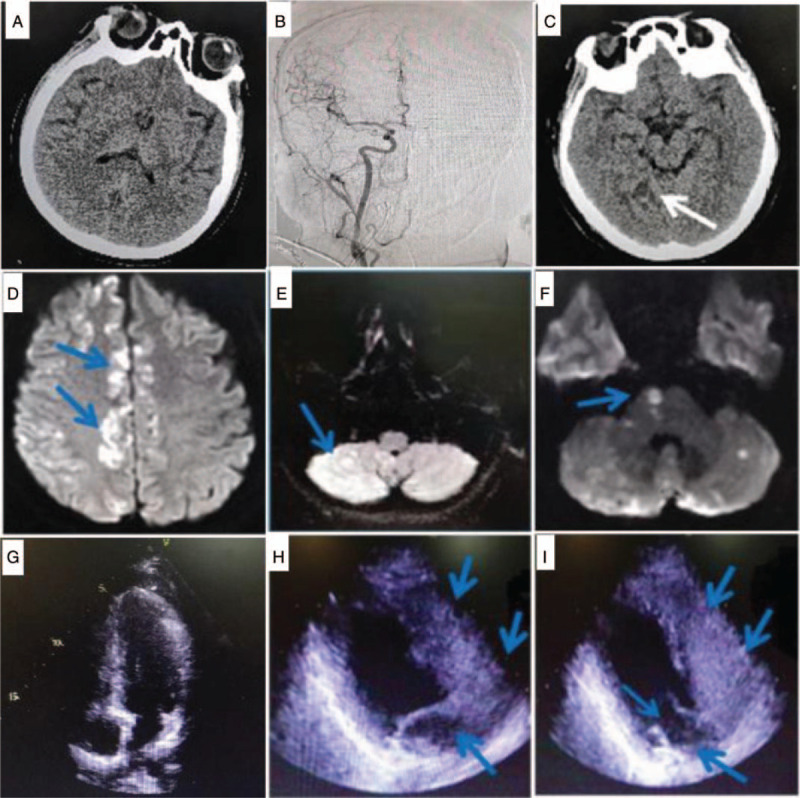
The imaging examination. A, An emergency CT examination was performed. No obvious abnormality was found in the density of brain parenchyma. B, DSA angiography showed no obvious abnormality in the whole cerebral blood vessel network (the image is the angiography image of the right carotid system). C, CT was rechecked 2 days after surgery, and the density of brain parenchyma was found to be uneven. On the right side, new patchy and slightly low-density shadows are seen in the basal ganglia and temporal occipital lobe. On the 5th day postsurgery, MR showed multiple lesions in bilateral brain, cerebellum, and brainstem, more pronounced on the right side; DWI, Diffusion Weighted Imaging images showed multiple spot-like, patch-like, line-like, and nodule-like high signal shadows in the bilateral cerebrum (D), cerebellum (E), and brainstem (F), more pronounced on the right side. G, The echocardiography reexamined after the operation showed that the structure of the heart was basically normal, no signal of left to right shunt was detected in the middle of the atrial septum; no echo interruption was detected, the ventricular septum was continuous and complete, and there was no sign of patent ductus arteriosus. H, Contrast echocardiography of the right heart. 10 days after the operation, normal saline contrast agent (agitated saline mixed with air) was injected by left elbow vein, the images of the right atrium and right ventricle were sequentially completed. And a small amount of contrast signal (blue arrow) appeared in the left atrium and left ventricle after 3 cardiac cycles. I, The above process is repeated twice, with identical results: in the image, the mitral valve has opened, and a small amount of contrast signal has entered the left ventricle. DSA = digital subtraction angiography, DWI = diffusion weighted imaging, MR = magnetic resonance.

The patient was admitted to the post anesthesia care unit (PACU) and resumed regular spontaneous breathing (tidal volume > 400 mL, respiratory rate 16 times/min), was deoxidized for 10 min (SpO_2_ > 95%), and was then extubated after an intravenous injection of atropine 0.5 mg+ neostigmine 1 mg. After extubation, the patient had stable vital signs and normal spontaneous breathing, but woke up only after 2 hours, gazing to the left. She was unable to communicate, with poor recovered consciousness and frequent vomiting. Blood gas analysis in PACU: pH = 7.37, PaCO_2_ = 38 mm Hg, PaO_2_ = 114 mm Hg, K^+^ = 3.5 mmol/L, Ca^2+^ = 1.16 mmol/L, HCO_3_^-^ = 21.4 mmol/L, Glucose = 6.4 mmol/L, Base Excess = −3.4 mmol/L, Hb = 128 g/L, SaO_2_ = 98%. The patient was given 10 mg metoclopramide intravenously for nausea and vomiting. In terms of recovery of consciousness, the patient exhibited a frowning reflex on the right side of the face in response to the noxious stimuli, while the observed left facial movement was less, attracting the attention of the perioperative physician. The anesthesiologist believed that this was not an ordinary case of delayed awakening, and communicated with the urologist and asked the neurologist for an urgent consultation. The patient's left upper limb movement was poor, the left lower limb could not move, and the left Babinski sign was positive; thus, cerebral hemorrhage or infarction was suspected. Subsequently, a noncontrast head computed tomography (CT) in emergency was performed, with no obvious abnormality found in the brain parenchymal density (Fig. 1A), thus excluding cerebral hemorrhage. According to the signs and symptoms, acute cerebral infarction with intracranial artery occlusion was hypothesized. A further digital subtraction angiography was performed in the department of vascular intervention, to prepare for interventional catheter thrombectomy and thrombolysis. Unexpectedly, no clear embolism was identified in the whole cerebrovascular system (Fig. 1B). According to the signs and symptoms, the patient was initially diagnosed as acute life-threatening brain stem infarction and was transferred to intensive care unit, due to the possibility of sudden death because of compression in the medulla oblongata cardiovascular center during the peak period of cerebral edema after brain stem infarction. The patient was intubated again due to hypoxemia (SpO_2_ 89%). CT re-examination on the second postoperative day revealed only that the density of brain parenchyma was less uniform than before, and a new patchy, low-density shadow was observed in the right basal ganglia and temporal occipital lobe (Fig. 1C). The patient did not show any improvement, despite receiving neuroprotective drugs (Vinpocetine, Edaravone, and Xingnaojing, which are commonly used as a standard of care in China), antiplatelets and other symptomatic treatments, for example, mannitol dehydration treatment to reduce intracranial pressure, sedation, anti-infection, plus dexamethasone to relieve edema at the same time. The patient subsequently awoke, with her left limb movement showing obvious signs of recovery, at which point the doctors decided to transfer her to another hospital for hyperbaric oxygen therapy.

The patient recovered consciousness on the third postoperative day. She was able to move her right limb according to instructions; the left upper limb had less pain-stimulus activity, while the left lower limb had no obvious pain-stimulus activity. The left Babinski sign was still positive. To further clarify the cause, a subsequent brain MRI examination was carried out when the patient's vital signs were stable, which showed a diffuse macular shadow of the whole brain, with gas embolism the suspected cause (Fig. 1D–F). On the seventh day after surgery, the movement of left upper and lower limbs was obviously improved, muscle strength was grade 5, and the left positive Babinski sign had disappeared.

It is worth mentioning that at the time of cerebral infarction, the patient also suffered from myocardial ischemia injury. The postoperative biomarker N-terminal pro-brain natriuretic peptide, and myocardial injury markers troponin I and creatine kinase MB also increased, as shown in Table 1.

**Table 1:** Postoperative changes of myocardial biomarkers in the patient.

Cardiac biomarkers (reference range)	Day 1	Day 2	Day 3	Day 4	Day 5	Day 6
NT-proBNP, pg/mL (12–133)	70	1060	2120	1350	339	238
TnI, ng/mL (0.01–0.023)	0.110	0.900	0.250	0.110	0.064	0.031
CK-MB, ng/mL (0.1–4.94)	10	40	2.80	<2.0	2.10	3.30

CK-MB = creatine kinase MB, NT-proBNP = N-terminal pro-brain natriuretic peptide, TnI = troponin I.

According to the images from the brain magnetic resonance examination, it was concluded that the cerebral infarction was caused by gas embolisms. To further determine the cause, transthoracic echocardiography was re-examined after the patient had recovered, and no obvious atrial septal defect, ventricular septal defect, or patent ductus arteriosus was identified (Fig. 1G). Following this, a right heart contrast-enhanced echocardiography was performed, the results of which were consistent with the sonographic findings (Fig. 1H, I) of a potential patent foramen ovale (PFO). The diagnosis was clear considering that the cerebral infarction was caused by gas embolisms passing through the PFO channel. The patient was hospitalized for 14 days and discharged after recovery. The patient was diagnosed as having right renal clear cell carcinoma according to the postoperative pathological report. At the latest follow-up in August 2019, the patient was in good health without obvious sequelae.

## 3 Discussion

According to the literature, cerebral infarction caused by air embolisms in the perioperative period is very rare.^[9]^ Carbon dioxide embolization following vascular injury in minimally invasive endoscopic surgery is also a rare but often fatal complication,^[8]^ generally accompanied by pulmonary embolism and cardiovascular collapse. If identified in time, the change will be made to open surgery. Successful rescue will not lead to further serious neurological complications. One widely described mechanism of gas embolism is venous embolic occlusion due to retrograde upward migration of gas bubbles; but the possibility is invalid, as the left lateral decubitus position was used for our patient, and the head position was not higher than the right atrium. In the initial CT images, there was no evidence of bubbles in the cerebral sinuses. We believe that PFO is the most likely way for the gas bubbles to enter the arterial system, which was later confirmed by a right heart contrast echocardiography. In this case, due to the potential PFO, a massive amount of carbon dioxide gas embolisms did not block the lungs, but rather directly entered the left heart through the foramen ovale, resulting in acute cerebral infarction. This consequence was difficult to diagnose, because invasive blood pressure monitoring during the procedure was very stable, there was no obvious circulatory abnormality or change of ETCO_2_, and only an unexpected transient fluctuation of the heart rate (45–120/min). According to the research literature, the reduction of ETCO_2_ is often the first sign of gas embolism, which generally decreases by more than 30%, and around half of patients will have some degree of cardiovascular event.^[8]^ In this case, however, there was no obvious change in ETCO_2_. From the observation of vital signs, this was an occult cerebral infarction with gas embolisms, which is difficult to identify.

In our patient the final clinical outcome was favorable. In this paper, we show that the reason for the delayed recovery of patients identified in the PACU is not the residue of anesthetics, but rather cerebral infarction caused by carbon dioxide gas embolisms, which is extremely rare, manifested as lethal brainstem infarction. PFOs may be the underlying cause in about 40% of cryptogenic ischemic strokes reported in the literature.^[10,11]^ We emphasize that the cause of postoperative coma may be cerebral infarction and paradoxical carbon dioxide gas embolism, in which a PFO is the pathogenesis. In this case, under the pneumoperitoneum pressure of 20 mm Hg, a large amount of carbon dioxide was absorbed into the circulation with the breach of the vena cava, entered the left heart through the potential PFO and was distributed to all organs. Diffuse gas embolisms blocked the endings of cerebral blood vessels, and the right brain injury was more serious as the patient was lying in left lateral decubitus position. It was in fact a case of whole-brain diffuse gas embolisms, although it was manifested as brainstem infarction. The patient gradually returned to normal 10 days after the operation, indicating that the prognosis was relatively good after the absorption of the carbon dioxide gas embolisms, and no obvious sequelae remained. It is reported that PFOs exist in about 25% to 35% of adults,^[12]^ so once there is a vein rupture or break in this kind of patient, there will be a large amount of carbon dioxide gas absorbed into the circulation under a certain level of pneumoperitoneum pressure, which will directly enter the left heart through the foramen ovale, damaging major organs such as the brain, while there is no manifestation of pulmonary embolism. It also seems to suggest that pneumoperitoneum surgery may lead to serious complications in case of a ruptured vein, as the presence of a PFO is difficult to identify (by echocardiography preoperatively, or even by bedside transthoracic ultrasound postoperatively).

It is necessary to discuss whether it is worth continuing pneumoperitoneum surgery after vascular rupture, as target organs, for example, heart, lungs, and brain may suffer various degrees of injury. From the serious consequences in this case, of course, pneumoperitoneum should be ceased. Pneumoperitoneum pressure may cause gas to flow back into the vena cava and right atrium from the small veins; and in this case, the vena cava injury also increased the level of risk. But if there is vascular injury, must there necessarily be a switch to laparotomy? It has been reported that laparoscopic suturing of blood vessels as soon as possible can minimize the harm caused by air embolism, and it is not necessary to switch to laparotomy,^[13]^ but the advantages and disadvantages need to be weighed. This case of cerebral infarction with gas embolism is occult, so it suggests that it is very important for a rapid diagnosis in this kind of situation. We think it is necessary to monitor transoesophageal echocardiography during this kind of surgery. If routine monitoring cannot be realized, at least pneumoperitoneum should be stopped when this difficult situation occurs; at the same time, transoesophageal echocardiography (TEE) intervention must be rapid for the diagnosis. Once paradoxical gas embolism is detected, pneumoperitoneum should be stopped and a switch made to laparotomy in time for hemostasis to avoid serious life-threatening complications in the cardiovascular and cerebrovascular systems. If there is no serious gas embolism, surgery under pneumoperitoneum can continue to ensure patients get the best benefit-risk ratio by avoiding laparotomy. TEE is a gold standard method, which can detect small bubbles of 5 to 10 μm at a concentration of 0.02 mL/kg.^[14]^ The routine application of TEE can provide an early diagnosis of gas embolism and prevent it from further developing into clinically significant cardiovascular and cerebrovascular events.^[15]^ In any case, when more gas emboli are identified, pneumoperitoneum should be stopped and switched to laparotomy, and the patient placed in the Trendelenburg position to prevent further damage to brain cells.

For this patient, the most likely explanation for the paradoxical gas embolism is the presence of a PFO. In this case, a contrast-enhanced echocardiography of the right heart was performed, the results of which were consistent with the sonography of a PFO. Because we identified that there were bubbles entering the left heart after 3 cardiac cycles, while we did not detect the PFO in the preoperative and postoperative transthoracic ultrasounds in this case, it cannot be ruled out that the PFO would only have opened under pneumoperitoneum pressure.

Another mechanism may exist. The explanation is that when a large amount of carbon dioxide enters the circulation, it exceeds the filtering effect of the lungs,^[16]^ overflows into the left heart circulation, and impacts the brain cells. There is a report in the literature of circulatory failure caused by paradoxical gas embolism in the case of hepatic vein rupture, although there is no right to left shunt, and TEE detected bubbles in the left heart.^[17]^ Under normal circumstances, the small bubbles that reach the pulmonary circulation will diffuse to the alveoli and be exhaled, preventing the gas from entering the left heart. However, the pulmonary circulation has a filter threshold for gases; once this threshold is exceeded, microbubbles will bypass the pulmonary circulation and be detected in the left heart.^[1]^ It is worth noting that CO_2_ is a relatively safe gas, for example, a small amount of CO_2_ bubbles’ entering has no clinical significance, unless it impacts important organs, for example, embolizing coronary or cerebrovascular arteries as in this case, causing not only cerebral infarction, but also causing cardiac biomarkers to rise.

## 4 Conclusions

As far as we know, this is the first case of cerebral infarction caused by diffuse paradoxical CO_2_ gas embolism identified due to severe delayed recovery after retroperitoneal laparoscopic nephrectomy. This kind of dangerous gas embolism leading to cerebral infarction can occur quietly while vital signs are stable. The relevant pathophysiological changes need to be further analyzed to find the best treatment. When encountering the situation of blood vessel injury or rupture, the decision whether to continue pneumoperitoneum or convert to open surgery for hemostasis needs to take into account the risk-benefit ratio of patients. Anesthesiologists and surgeons should be aware of the risk of hidden catastrophic complications and maintain timely communication during the perioperative period. The method of treatment will vary on an individual basis, depending on differences in patients’ anatomy, but at such junctures the advantages and disadvantages should be weighed promptly and decisively to avoid serious systematic complications.

## Acknowledgments

The authors thank the National Natural Science Foundation of China (81500178) and the Hospital Medical Research Foundation of Peking University Third Hospital (NO. BYSY2016004) for the support. Thanks to Richard Lester from Peking University Health Sciences Center for help in editing and polishing the language of this paper.

## Author contributions

M.X. analyzed and interpreted the patient data regarding treatment details and images. Y.Y. performed the data acquisition and literature review, and was a major contributor in writing the manuscript. All authors read and approved the final manuscript.

**Conceptualization:** Youxiu Yao, Mao Xu.

**Data curation:** Mao Xu.

**Funding acquisition:** Youxiu Yao, Mao Xu.

**Investigation:** Youxiu Yao.

**Supervision:** Mao Xu.

**Writing – original draft:** Youxiu Yao.

**Writing – review & editing:** Youxiu Yao, Mao Xu.
